# Resource Allocation during an Influenza Pandemic

**DOI:** 10.3201/eid1403.071275

**Published:** 2008-03

**Authors:** Karthikeyan Paranthaman, Christopher P. Conlon, Claire Parker, Noel McCarthy

**Affiliations:** * Public Health Department, Oxford, UK; † John Radcliffe Hospital, Oxford, UK; ‡ Jericho Health Centre, Oxford, UK; § Thames Valley Health Protection Unit, Oxford, UK

**Keywords:** disease outbreaks, human influenza, resource allocation, clinical protocols, population surveillance, letter

## Abstract

Resource Allocation during an Influenza Pandemic

**To the Editor:** Planning for pandemic influenza is accepted as an essential healthcare service and has included creation of national and international antiviral drug stockpiles and novel approaches to emergency vaccine development (*1*). The effectiveness of these strategies in a pandemic may be substantial but is unknown. More certain is that effective management of severe and complicated influenza will reduce deaths and that demand will exceed available treatment resources (*2*). Appropriate allocation of treatment resources is therefore essential, perhaps more important than any specific treatment such as administering antiviral medication to symptomatic patients. Resource allocation requires the following: 1) making clear societal decisions on the goals for healthcare resources; 2) conducting operational research to develop an evidence base to support the achievement of these goals; and 3) developing systems to capture and learn from new information in a pandemic to facilitate modification of the response as the characteristics of the pandemic emerge. Most societies have not yet addressed the first issue fully. In the United Kingdom, a Department of Health consultation on planning critical care during emergencies cites “the underpinning principle of providing the greatest good for the greatest number of people during the course of an emergency” and thus appears to support a capacity-to-benefit approach (*3*). Similarly, triage criteria developed in Canada, based on the Sequential Organ Failure Assessment (SOFA) score, exclude those persons believed to be too ill or otherwise unlikely to benefit from critical care (*4*). Even more important for most severely ill patients, however, will be deciding whether to admit them to the hospital at all. The UK pandemic-planning criteria currently recommend a scoring system for hospital admission based on an assessment of poor outcome rather than on capacity to benefit (*2*). Indeed, age >85 years and severe underlying cognitive impairment, which would rule out admission to critical care in Canada, would strongly favor admission to hospital care in the United Kingdom, the opposite of the situation for a younger cognitively intact person with similar disease severity. If tools are to be developed to support triage at all stages of the patient pathway in a pandemic, societies must consider the ethical issues raised (*4*,*5*), debate them, and take a position on the values that should underpin decision making in a pandemic.

Even when clear societal goals are established, much work remains to ensure that the healthcare community is equipped to steer healthcare resources to deliver these effectively (*6*). Community-acquired pneumonia has been used as a surrogate for influenza to test predictive scoring systems for assessing severity and assisting triage decisions (*7*). Seasonal influenza epidemics would provide the most realistic setting available, in particular, if protocols were in place to test criteria when a relatively severe influenza season occurs. In addition to identifying criteria for setting priorities within influenza management, such testing will need to consider the balance of resources between influenza treatment and treatment of other usual noninfluenza conditions that will require emergency care during the pandemic. Decisions that must be made during a pandemic are complex, varying from when to stop major elective surgery so critical care capacity can be opened up, to how to triage those who have experienced major trauma and those with influenza. These decisions could differ from those same decisions made outside a pandemic, and an adequate evidence base is needed if they are to be of good quality.

The third component of our preparation for optimally deploying standard care in a pandemic is being able to change our approach quickly as new knowledge emerges. In the so-called Spanish influenza pandemic of 1918–19, the unfamiliar clinical course meant that influenza was not even considered when the first cases appeared (*8*), and expectations had to be revised concerning who was most vulnerable and at what stage in their clinical course they were most at risk. Therefore, healthcare professionals must develop and test the public health infrastructure to capture patient factors associated with outcome and treatment response during a pandemic and feed this information back into clinical practice rapidly and reliably, as occurred during the epidemic of severe acute respiratory syndrome (*9*). International collaboration will be important for sharing this work (*10*) and developing useful tools early in a pandemic. Having recognized the risk for pandemic influenza, we must now complement the research into novel influenza treatments by addressing our knowledge gap on how best to use our resources to deliver optimal clinical care in the management of influenza guided by effective clinical surveillance.

## References

[1] Mounier-Jack S, Coker RJ. How prepared is Europe for pandemic influenza? Analysis of national plans. Lancet. 2006;367:1405–11. 10.1016/S0140-6736(06)68511-5 16650650

[2] Health Departments UK. Pandemic flu: UK influenza pandemic contingency plan. October 2005 [cited 2007 Nov 20]. Available from http://www.dh.gov.uk/assetRoot/04/12/17/44/04121744.pdf

[3] Health Departments UK. The NHS emergency planning guidance 2005: underpinning materials. Critical care contingency planning in the event of an emergency where the number of patients substantially exceeds normal critical care capacity. Best Practice Guidance. August 2006 [cited 2007 Nov 20]. http://www.dh.gov.uk/assetRoot/04/13/76/19/04137619.pdf

[4] Christian MD, Hawryluck L, Wax RS, Cook T, Lazar NM, Herridge MS, Development of a triage protocol for critical care during an influenza pandemic. CMAJ. 2006;175:1377–81. 17116904 10.1503/cmaj.060911 PMC1635763

[5] Torda A. Ethical issues in pandemic planning. Med J Aust. 2006;185(Suppl):S73–6. 17115958 10.5694/j.1326-5377.2006.tb00713.x

[6] Reilly BM, Evans AT. Translating clinical research into clinical practice: impact of using prediction rules to make decisions. Ann Intern Med. 2006;144:201–9. 16461965 10.7326/0003-4819-144-3-200602070-00009

[7] Challen K, Bright J, Bentley A, Walter D. Physiological-social score (PMEWS) vs. CURB-65 to triage pandemic influenza: a comparative validation study using community-acquired pneumonia as a proxy. BMC Health Serv Res. 2007;7:33. 10.1186/1472-6963-7-33 17328822 PMC1819377

[8] World Health Organization. Avian influenza: assessing the pandemic threat. January 2005 [cited 2007 Nov 20] Available from http://www.who.int/csr/disease/influenza/H5N1-9reduit.pdf

[9] Leung GM, Rainer TH, Lau FL, Wong IO, Tong A, Wong TW, A clinical prediction rule for diagnosing severe acute respiratory syndrome in the emergency department. Ann Intern Med. 2004;141:333–42. Epub 2004 Aug 23. 15326019 10.7326/0003-4819-141-5-200409070-00106

[10] Naylor CD, Chantler C, Griffiths S. Learning from SARS in Hong Kong and Toronto. JAMA. 2004;291:2483–7. 10.1001/jama.291.20.2483 15161900

